# Editorial: 3D Modelling of Mammalian Embryos and Organs

**DOI:** 10.3389/fcell.2021.763008

**Published:** 2021-09-22

**Authors:** Silvia Garagna, Elisa Cebral, Juan Aréchaga, Maurizio Zuccotti

**Affiliations:** ^1^Department of Biology and Biotechnology Lazzaro Spallanzani, University of Pavia, Pavia, Italy; ^2^Departamento de Biodiversidad y Biología Experimental, Facultad de Ciencias Exactas y Naturales, Universidad de Buenos Aires, Buenos Aires, Argentina; ^3^Department of Cell Biology and Histology, School of Medicine and Nursing, University of the Basque Country, Leioa, Spain

**Keywords:** embryos, organs, organogenesis, organoids, 3D imaging, 3D modeling, gastruloids

The main scope of this Special issue was to gain understanding on how tissues and organs are arranged into integrative hierarchical levels of complexity, from the molecular to the morphological organization. To understand the underlying complexity of the relationships among these levels during morphogenesis or in the adult we must learn how to resolve single-cell spatial relationships in the three-dimensional (3D) organization of tissues, organs, and even of the whole organisms.

Each paper in this “Frontiers” special issue contributes to deepen our understanding on the dynamics and mechanisms of development and differentiation in the complexity of the 3D architecture of embryos, intact organs, or organoids.

Five out of 13 contributions, which include Original articles, Reviews, Perspectives, Hypothesis and Theories, focused on intact tissues or organs. In the other eight papers, 3D stem cell culture systems, coupled with advanced microscopy approaches, are exploited to recapitulate morphogenesis and differentiation with the creation of gastruloids, spheroids, or organoids where the cellular organization and function of specific tissues and organs are mimicked. A main content of each contribution is briefly described below starting with those papers focusing on organs and then with those that have used stem cell cultures in 3D.

The use of focused-ion beam-scanning electron microscopic (FIB-SEM) tomography applied to resin-embedded rat adult cortex kidney, allowed Kawasaki et al. to reconstruct, with unprecedented accuracy, the first ultrastructural 3D organization of glomerular endothelial cells (GEnCs). In their paper, entitled “Three-Dimensional Architecture of Glomerular Endothelial Cells Revealed by FIB-SEM Tomography,” these cells are made of three major subcellular compartments -the cell body, cytoplasmic ridges, and sieve plates—in addition to the globular protrusions and the reticular porous structures). Moreover, GEnCs organize, without obvious joints, in a tubular shape. This paper reveals the mystery of the capillaries of the rat kidney and sets the bases for 3D structural studies on the glomerular capillary system during development, in physiological or pathological conditions and in different species.

In their elegant study “Three-Dimensional Analysis of Busulfan-Induced Spermatogenesis Disorder in Mice” Nakata et al. adopted a conventional histological approach to perform a 3D reconstruction of all the seminiferous tubules present in the mouse testis treated with busulfan to impair spermatogenesis. They found that impaired tubule segments increased in length, but not in number, in a dose-dependent manner and that the severity of busulfan-induced spermatogenesis disorder is not fixed in location among individuals.

The architecture of intact organs at the single cell level was addressed in two articles where limitations of traditional histology were overcome by the use of an iDISCO-based clearing approach coupled with light-sheet microscopy or micro-computed tomography.

In their paper “3D reconstruction of the clarified rat hindbrain choroid plexus” Perin et al. reconstructed the rat hindbrain choroid plexus macro-and microstructure, using markers for epithelium, arteries, microvasculature, and macrophages, and found its association with 4th ventricle-related neurovascular structures.

On formalin-fixed intact ovaries, Fiorentino et al. in their “Three-Dimensional Micro-Computed Tomography of the Adult Mouse Ovary” determined the number of follicles, from pre-antral secondary to antral, of *corpora lutea*, and described the major vasculature, defining their precise 3D localization within the organ volume. They suggest that follicle recruitment is homogeneously distributed all-over the cortex and that follicles grow within the less stiff environment of the medulla until when they re-emerge on the cortex surface of the same tissue region, ready to be eventually ovulated.

The importance of a 3D analysis of the ovary is well-underlined in the Review “Ovary Development: Insights From a Three-Dimensional Imaging Revolution” where Soygur and Laird stress how 3D modeling has evidenced new relationships and levels of organization among follicles during the dynamic tissue remodeling at each hormonal cycle and, also, suggest to exploit ovarian architecture toward therapeutic applications.

Ismagulov et al. in their inspiring Review entitled “Epithelial-Mesenchymal Transition Drives Three-Dimensional Morphogenesis in Mammalian Early Development” present a model in which cellular morphogenesis is the driving force of embryonic 3-D organization. They suggest that the use of *in vitro* platforms where cells grow and differentiate on biomaterials capable of dynamic modulation of their physical properties will make possible to study the integration between mechanical signals and downstream effectors of pathways leading to cell specification and tissue patterning.

The importance of a 3D modeling approach extended to organoids is nicely summarized by Susaki and Takasato in their Perspective “Extending the Utility of Three-Dimensional Organoids by Tissue Clearing Technologies” which contributes with a wide panorama of basic research applications on organoids and provides a toolbox for the study of 3D alterations occurring during either drug administration or deviation from homeostasis to pathology.

On the same line, Kwon et al. in their Review “Intestinal Morphogenesis in Development, Regeneration, and Disease: The Potential Utility of Intestinal Organoids for Studying Compartmentalization of the Crypt-Villus Structure” explore how the current technology of 3D intestinal organoids contributes to the understanding of the regulatory factors and signaling pathways acting during development, maintenance, and regeneration of crypt-villi in the intestinal epithelium.

Organoids are also an innovative technique to study human brain development, but limitations due to heterogeneity and long-term differentiation still hinder their application in disease modeling and drug discovery. In their study “Simplified Brain Organoids for Rapid and Robust Modeling of Brain Disease,” Ha et al. developed homogeneous simplified brain organoids generated in only 2 weeks and made hPSC-derived mature neurons and astroglial cells that can be applied to large-scale disease models and alternative drug-testing platforms.

A rapid and robust protocol to induce neural rosette formation from monolayer cultures of human pluripotent stem cell-derived neural progenitor cells (NPC) was developed by Townshend et al. and described in their original article “Effect of Cell Spreading on Rosette Formation by Human Pluripotent Stem Cell-Derived Neural Progenitor Cells.” The Authors by demonstrating that NPC rosette morphogenesis requires basal spreading activity tightly regulated by Rho/ROCK signaling, provide a cell platform for further investigations on the cellular and molecular mechanisms underlying NPC rosette morphogenesis.

Telencephalon patterning was investigated by Nasu et al. in their “Two-Phase Lineage Specification of Telencephalon Progenitors Generated From Mouse Embryonic Stem Cells” using a 3D *in vitro* differentiation embryonic stem cell model, verifying the contribution of the ventralizing Shh, the dorsalizing BMP and WNT signals. The study reveals a novel developmental mechanism that depends on a dose- and time-dependent signaling interplay which includes a time lag for specification and a temporal shift in cellular Shh sensitivity.

By differentiating patient-derived microtic cartilage stem/progenitor cells using a 3D spheroid culture platform, Zucchelli et al. in “Modeling Normal and Pathological Ear Cartilage *in vitro* Using Somatic Stem Cells in Three-Dimensional Culture” showed that microtic spheroids were asymmetric, hyper-cellularized, the inner, and outer regions did not develop properly with an organization similar to that of native microtic cartilage. Importantly, they observed that microtic cartilages are vascularized. The use of a 3D self-organizing *in vitro* system allowed to identify novel features of microtic ears demonstrating the value of this type of approach for both understanding differentiation and for disease modeling.

Lung epithelial morphogenesis and differentiation were studied by Rabata et al. and described in their paper “3D Cell Culture Models Demonstrate a Role for FGF and WNT Signaling in Regulation of Lung Epithelial Cell Fate and Morphogenesis” employing mouse lung epithelial stem/progenitor cells differentiated in lungospheres (in non-adherent cultures) or lung organoids (using a 3D extracellular matrix culture system). The Results obtained from the two different culture systems revealed distinct roles for FGF ligands in regulating lung morphogenesis and differentiation.

Overall, we believe that the topic of this collection of papers is both relevant and timely. Although limited to specific examples, it overwhelmingly emerges that a significant understanding of development, differentiation, homeostasis, or pathogenesis will be possible when these processes will be studied in the 3D organization of the biological structures.

## Author Contributions

All authors listed have made a substantial, direct and intellectual contribution to the work, and approved it for publication.

## Conflict of Interest

The authors declare that the research was conducted in the absence of any commercial or financial relationships that could be construed as a potential conflict of interest.

## Publisher's Note

All claims expressed in this article are solely those of the authors and do not necessarily represent those of their affiliated organizations, or those of the publisher, the editors and the reviewers. Any product that may be evaluated in this article, or claim that may be made by its manufacturer, is not guaranteed or endorsed by the publisher.

